# Stacking Interactions in Indomethacin Solid-State Forms

**DOI:** 10.1021/acs.cgd.4c01507

**Published:** 2025-03-10

**Authors:** Nazanin Fereidouni, Marwah Aljohani, Andrea Erxleben

**Affiliations:** † School of Biological and Chemical Sciences, University of Galway, Galway H91TK33, Ireland; ‡ Department of Chemistry, College of Science, Imam Abdulrahman Bin Faisal University, P.O. Box 76971, Dammam 31441, Saudi Arabia; § Synthesis and Solid State Pharmaceutical Centre (SSPC), Limerick V94 T9PX, Ireland

## Abstract

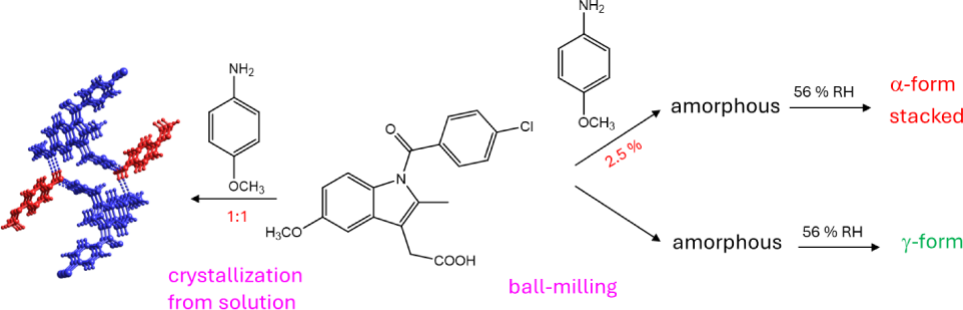

Stacked structures with strong dispersion forces between stack neighbors often lead to anisotropic crystal growth and needlelike morphologies. The crystal structures of a new cocrystal and a molecular salt of indomethacin (IND) are reported: IND·MOA and IND·POBA·0.5H_2_O (MOA = *p*-methoxyaniline, POBA = 4-phenoxybenzylamine). In both structures, the IND and coformer molecules/ions are stacked and IND adopts the unusual conformation found in the α-polymorph of pure IND, resulting in a relatively short distance of about 3 Å between the methyl group and the C1′-atom of the chlorophenyl ring. While IND·MOA and IND·POBA·0.5H_2_O both crystallize as needles like α-IND, the weaker stacking interactions of the coformer in the IND·MOA cocrystal lead to shorter and thicker needles. Amorphous IND prepared by milling recrystallizes to the stable γ-polymorph without the metastable α-form being detected. When IND is milled in the presence of 2.5 wt % MOA, the amorphous phase converts to α-IND. The effect of small amounts of the coformer on the recrystallization route is attributed to a templating effect of the cocrystal formed during milling and/or the facilitation of the conversion to the α-phase conformation.

## Abstract 2

Low levels of *p*-methoxyaniline affect the recrystallization pathway of milled, amorphous indomethacin, which is attributed to traces of cocrystal formation during milling.

## Introduction

Understanding the relationship between the structure and morphology of molecular crystals is of significant importance, both from a fundamental point of view and with a view to applications. The crystal shape has an effect on the mechanical and physicochemical properties such as compressibility, physical stability, bulk density and dissolution behavior with anisotropic shapes often giving undesirable material properties.^1−3^ There is a particular interest in the one-dimensional growth of needle-shaped molecular crystals which has been the topic of a large number of experimental and theoretical studies.^4−11^

Needle-like crystals form as a result of strong directional intermolecular interactions leading to fast growth in one direction.^12^ These interactions can be hydrogen bonding or van der Waals interactions. While van der Waals interactions are usually weaker per interaction than hydrogen bonding interactions, the total interaction energy from van der Waals interactions can be higher than that from hydrogen bonds just because of their larger number. We recently analyzed stacked structures and showed that those that maximize van der Waals contact stacking tend to grow as needles.^13,14^ This is particularly true, when there is a dominant stacking motif in the structure that has an interaction energy of more than 30 kJ mol^–1^ with more than 50% of the atoms in a molecule in van der Waals contact with their stack neighbor.^13^ We have proposed that dispersion forces are more effective for driving anisotropic crystal growth than hydrogen bonding because they obey a kr^–6^ law. Hydrogen bonding is Coulombic (kr^–2^ law) which attracts molecules from longer distances. If a molecule has more than one hydrogen bond donor/acceptor site it may be attracted to the growth site in the wrong orientation which may hinder crystal growth. The shorter distance range of dispersion forces makes the incorporation of a molecule in a wrong conformation or orientation less likely.^14^ It is important to emphasize that stacked structures are not limited to flat molecules. Nonflat molecules can form stacked structures and crystallize as long, thin needles if they self-fit.^14^ The α-polymorph of indomethacin is an example.^15^

**Figure 1:**
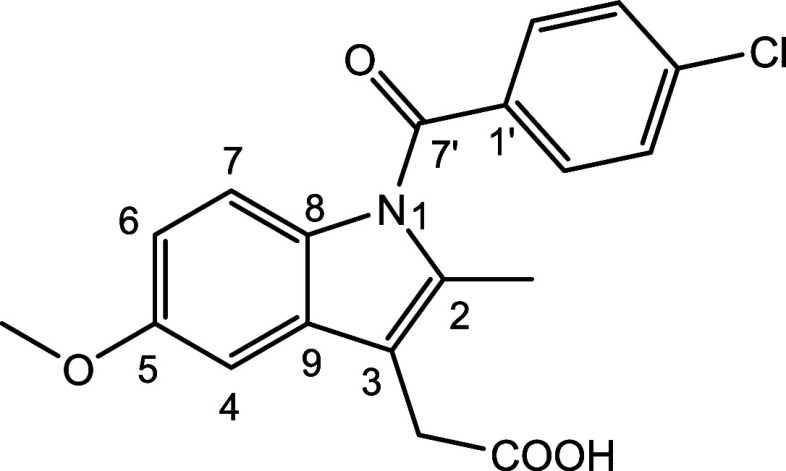
Chemical structure of indomethacin.

Indomethacin (IND, Figure 1) is a nonsteroidal anti-inflammatory drug used for the treatment of mild to moderate pain and the management of the symptoms of arthritis.^16^ It has seven known polymorphs, α, γ, δ, ε, η, τ, and ξ.^15,17−25^ The polymorphism and crystallization behavior of IND has been studied both experimentally and computationally by several research groups.^19,22,26−36^ The two most common polymorphs are the α- and γ-form. There has been general agreement that γ-IND is the thermodynamically stable form, while α-IND is metastable and that the two forms are monotropically related. However, the fact that α-IND has a higher crystallographic density than the γ-form (generally expected for the more stable polymorph) is somewhat puzzling and recently, Zeitler and coworkers reported a comprehensive study using experimental low-frequency vibrational spectroscopies, theoretical solid-state density functional theory and ab initio molecular dynamics calculations that challenged the accepted γ > α stability order.^26^

Eleven cocrystals of IND are reported in the Cambridge Structural Database to date.^37^ A cocrystal is a multicomponent crystal built up by two or more molecules that interact with each other through noncovalent interactions usually generating a crystal packing that is completely different from those of the parent molecules. The majority of the known IND cocrystals have a nonstacked structure.

Here we report a cocrystal and a molecular salt of IND that retain stacks of IND molecules/ions in their structure. We have analyzed the stacking interactions using Pixel and Indicative Lattice Analysis calculations and investigated the effect of the coformer on the total stacking interaction energy. We also observed that the presence of small amounts of coformer affect the recrystallization pathway of milled, amorphous IND.

## Experimental Section

### Materials

IND, *p*-methoxyaniline (MOA), and 4-phenoxybenzylamine (POBA) were purchased from Tokyo Chemical Industry (TCI Europe) and used as received. The solvents ethanol (Fisher Scientific) and ethyl acetate (Honeywell) were analytical grade and used without further purification.

### Solution Crystallization

#### IND·MOA

IND (35.7 mg, 0.1 mmol) and MOA (12.3 mg, 0.1 mmol) were dissolved in 10 mL of ethanol with stirring at 600 rpm and gentle heating. X-ray suitable single crystals of IND·MOA were obtained by slow evaporation of the solvent. IR (cm^–1^): 3355 m (ν(O–H)), 3285 w (ν(N–H)), 2921 w, 2839 w, 2048 w, 1978 w, 1669 s (ν(C=O), amide), 1599 m, 1513 m, 1464 s, 1358 s, 1286 s, 1219 s, 1140 s, 1077 s, 1022 s, 878 s, 823 s, 719 s.

#### IND·POBA·0.5H_2_O

IND (35.8 mg, 0.1 mmol) and POBA (59.7 mg, 0.3 mmol) were dissolved in 10 mL of ethanol. The solution was slowly evaporated at room temperature. Needle-shaped crystals formed after a few days. IR (cm^–1^): 3602 w (ν(O–H), water), 3431 w, 2885 w, 1669 m (ν(C=O)), 1593 m, 1542 s (ν(COO^–^), 1472 s, 1362 m, 1318 s, 1229 s, 1153 m, 1075 m, 1018 m, 838 s, 760 s.

### Ball-Milling

500–600 mg commercial IND or commercial IND physically mixed with 2.5 or 5 wt % MOA (Table S1) was placed in a 25 mL stainless steel milling jar containing a 15 mm (diameter) stainless steel ball and subjected to ball-milling at 25 Hz for 20 or 60 min using a Mixer Mill MM400 oscillatory ball mill (Retsch GmbH, Germany). Milling was conducted at room temperature with a 5 min cool-down period after every 15 min of milling. Commercial IND was confirmed by XRPD analysis to be the γ-polymorph.

Cryomilling of a physical mixture of IND and 0.5 mol equivalent MOA (total sample weight 586 mg) was performed by immersing the milling jar in liquid nitrogen for 5 min before milling for 60 min. Milling was performed in 10 min intervals with 5 min cooling periods in between. During cryomilling, the external temperature of the jars remained below −20 °C.

The milled samples were stored for 5 days in a desiccator at ambient temperature (20 ± 2 °C). A saturated solution of Mg(NO_3_)_2_ was used to maintain a relative humidity of 56%.^38^

### Infrared (IR) Spectroscopy

FT-IR spectra were collected in the 650–3600 cm^–1^ range using a PerkinElmer Spectrum 400 fitted with an ATR reflectance attachment with diamond/ZnSe optics. The resolution was 4 cm^–1^ and four integrated scans were used.

### Differential Scanning Calorimetry

Differential scanning calorimetry (DSC) analysis was conducted using a PerkinElmer DSC 4000. The samples were heated in closed aluminum crucibles at a rate of 10 °C/min from 20 to 300 °C.

### X-ray Powder Diffraction

X-ray powder diffraction (XRPD) analysis was performed on a Rigaku model Ultima IV diffractometer equipped with a Cu–K_α_ radiation source (λ = 1.54178 Å; 40 kV, 40 mA). Data were collected over the 5–50° range (2θ).

### Crystallography

The data collections for IND·POBA·0.5H_2_O and IND·MOA were performed at room temperature on an Oxford Diffraction Xcalibur system (Oxfordshire, UK) using graphite-monochromated Mo–K_α_ radiation (λ = 0.71073 Å). The structures were solved by intrinsic phasing using SHELXT^39^ and refined by full-matrix least-squares on F^2^ using SHELXL 2018/3^40^ within the Olex2 program suite.^41^ Non-hydrogen atoms were refined anisotropically. Further refinement was performed with olex2.refine with nonspherical atom scattering factors computed by NoSpherA2.^42^ Orca 5.0.2 was used to calculate the electron density at the PBE/def2-TZVP level of theory.^43^ The coordinates of all hydrogen atoms of IND·MOA were refined freely with isotropic atomic displacement parameters. Due to the low data: parameter ratio (resulting from the thin needle morphology) the hydrogen atoms of IND·POBA·0.5H_2_O were fixed in geometrically determined positions (except those of the disordered water molecule of crystallization that could not be placed) and refined as riding atoms with isotropic displacement factors equivalent to 1.2 times those of the atom to which they were attached (1.5 times for methyl groups). Drawings were made with ORTEX and POGL embedded in Oscail.^44^ Overlay plots were generated with UCSF ChimeraX.^45^ Crystallographic data and refinement details are provided in Table S2.

### Pixel Calculations and Hirshfeld Surface Analysis

Gavezzotti’s Pixel program^46^ embedded in the Oscail software package^44^ was used to calculate the intermolecular interaction energies and to partition them into Coulombic, polarization, dispersion and repulsion energies. The Pixel input was generated using ORCA/MultiWFN electron density with the 6-31G** basis set and the density functional theory functional wB97X-D3.^43,47^ The intermolecular energies in α-IND with *Z*’ > 2 were calculated in a pairwise fashion using PixelS.^44^

Hirshfeld surfaces and 2D fingerprint plots were generated from the cif files with CrystalExplorer 21.^48^

## Results and Discussion

### Needle Growth of α-Indomethacin

**Figure 2:**
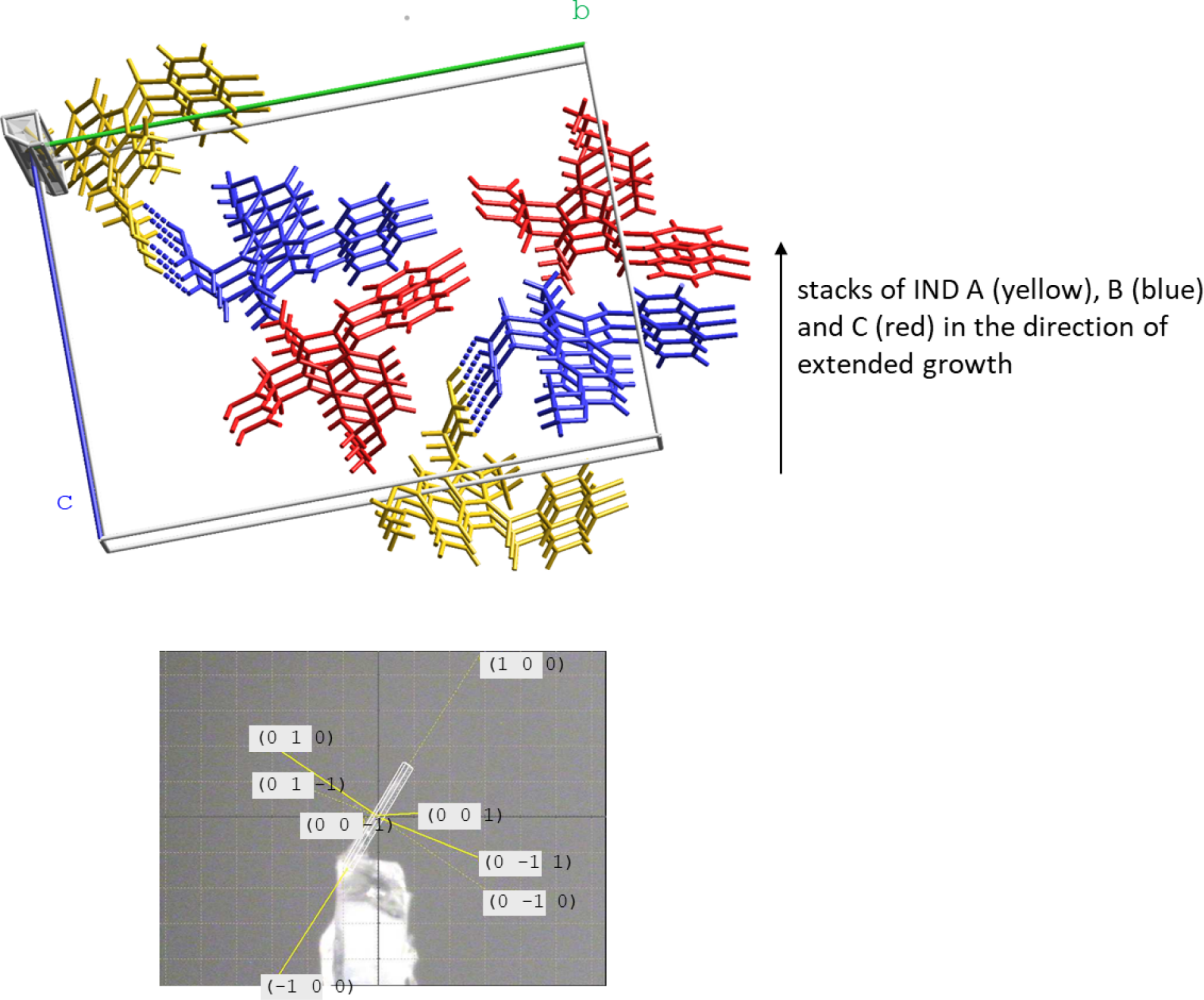
Stacked structure of α-IND (CSD ref code INDMET02^15^) and crystal growth along the *a* axis. Yellow: molecule A, blue: molecule B, red: molecule C.

The X-ray structure of needle-shaped α-IND crystals was reported by Stowell and coworkers in 2002.^15^ The monoclinic unit cell (space group P2_1_) contains three crystallographically independent molecules; A, B and C. A and B form the typical carboxylic acid dimer with pairwise C=O···HO hydrogen bonding. The carboxyl group of C forms a hydrogen bond with the amide oxygen of B. The A:::B···C entities stack along the *a* axis (Figure 2).

**Figure 3:**
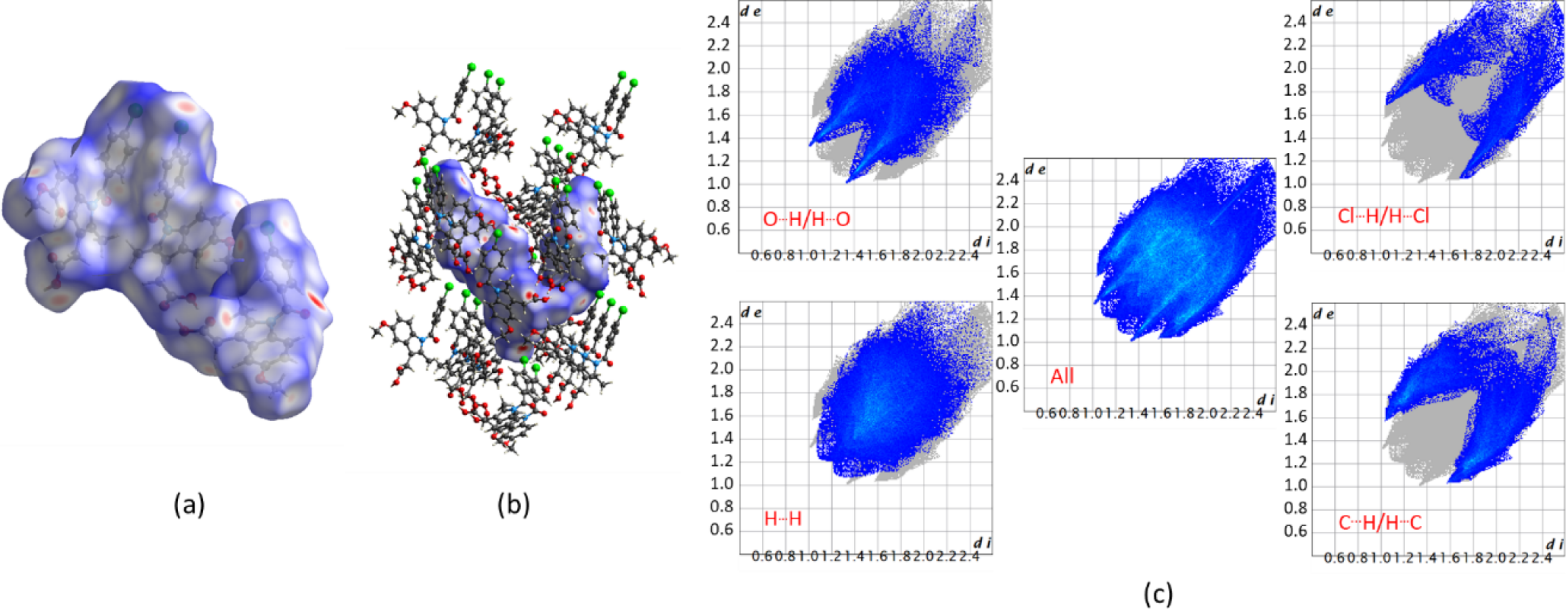
(a) Hirshfeld surface (*d*_norm_) of α-IND (CSD ref code INDMET02^15^). The red spots represent hydrogen bonding. (b) Molecules with atoms within a distance of 3.8 Å outside the Hirshfeld surface map and (c) 2D fingerprint plots (*d*_i_ vs *d*_e_, where *d*_i_ and *d*_e_ are the distances to the nearest atom inside and outside the surface, respectively.

To quantify the intermolecular interactions, the Hirshfeld surface map was generated (Figure 3). The 2D fingerprint plots show that the main contributions in the crystal packing are van der Waals H···H contacts (36.4%). C···H/H···C, O···H/H···O and Cl···H/H···Cl contributions are 21.9, 20.7 and 11.5%, respectively.

We have performed Indicative Lattice Analysis using the Interactive Lattice Analysis tool in the Oscail software package^44^ and Pixel calculations^46^ to rationalize the extended growth in the *a* direction. The Pixel program calculates intermolecular energies and allows to partition them into Coulombic, polarization, dispersion and repulsion energies. The strongest, the third strongest and the fourth strongest interaction in α-IND are dominated by dispersion (*E*_total_ = −104.1 kJ mol^–1^, *E*_dispersion_ = −69.5 kJ mol^–1^; *E*_total_ = −75.7 kJ mol^–1^, *E*_dispersion_ = −78 kJ mol^–1^; *E*_total_ = −65.3 kJ mol^–1^, *E*_dispersion_ = −75.1 kJ mol^–1^) and are the contact stacking interactions of B, C and A, respectively. The carboxyl dimer and C=O(amide)···HO hydrogen bonding interactions have interaction energies *E*_total_ of −75.8 and −34.4 kJ mol^–1^ with Coulomb contributions of −107.5 and −34.9 kJ mol^–1^, respectively. Details of the Pixel data are shown in Table S3. Within the stacks, 56, 57 and 60% of the atoms of IND A, B and C are in van der Waals contact with their stack neighbors, respectively. It is the efficient stacking with large interaction energies that drives the needle growth in line with our previous findings on nonflat molecules with stacked structures.^13,14^

### Crystal Structures of the Indomethacin-4-Methoxyaniline Cocrystal and the Indomethacin-4-Phenoxybenzylamine Salt

**Figure 4:**
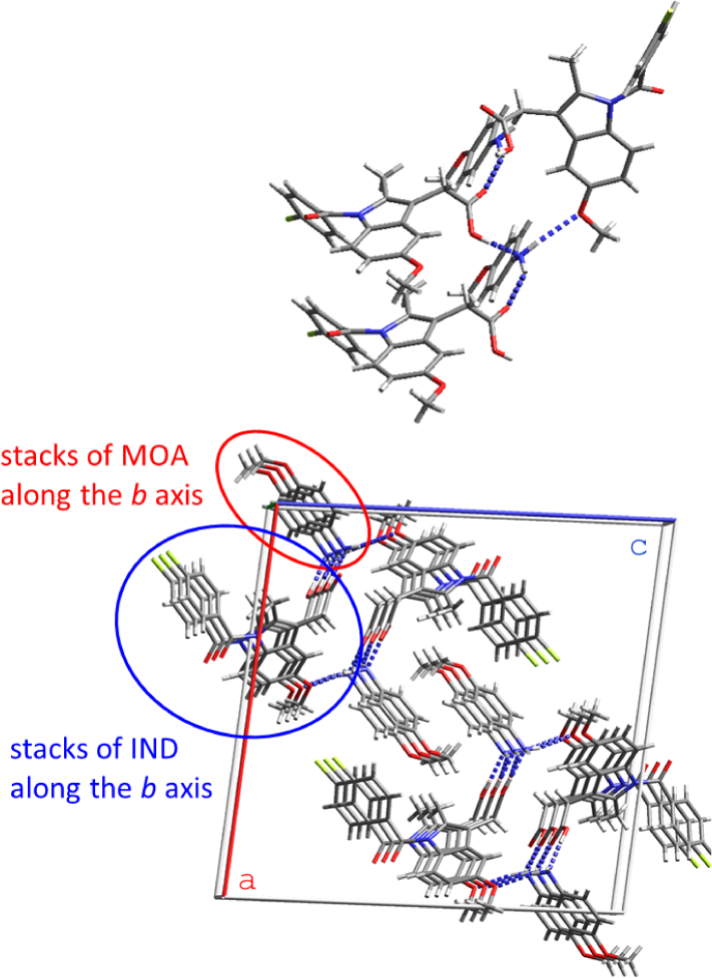
Hydrogen bonding motif and stacking in IND·MOA.

**Figure 5:**
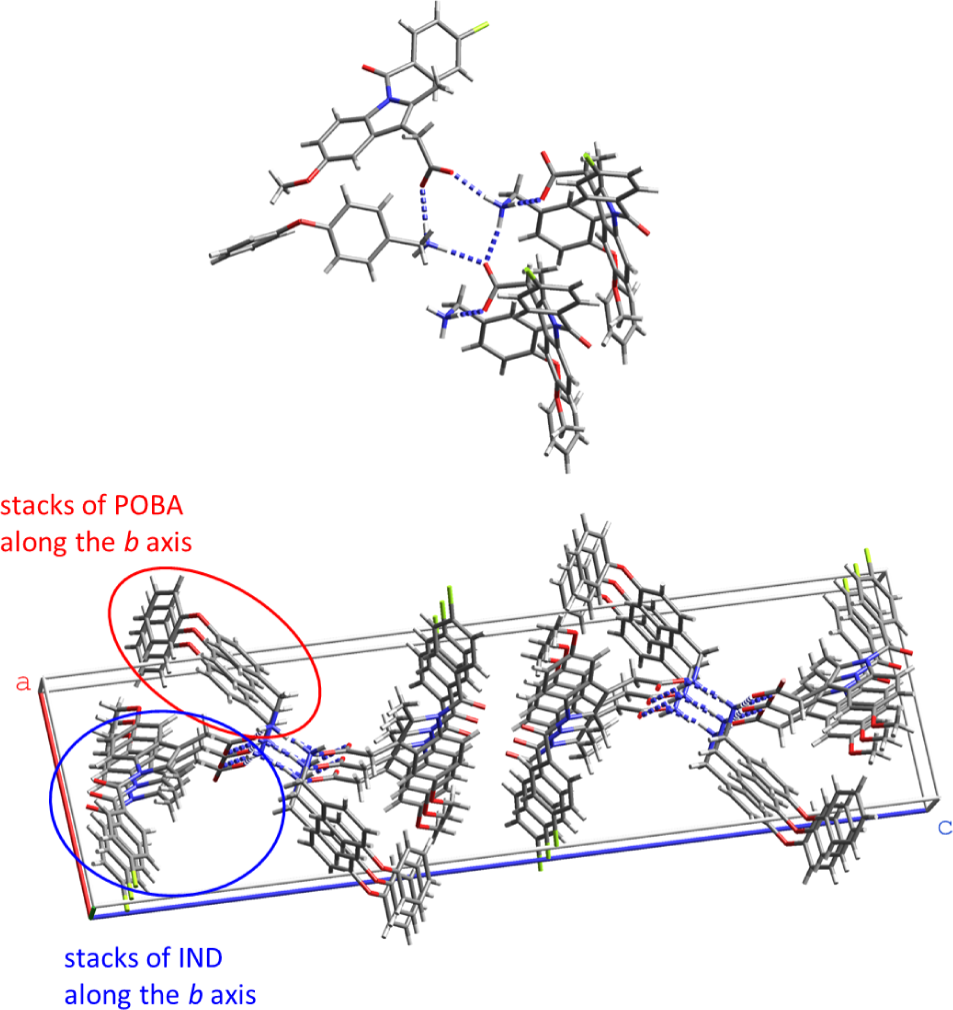
Hydrogen bonding motif and stacking in IND·POBA·0.5H_2_O. The water molecules of crystallization are not shown for clarity.

The 1:1 cocrystal of IND and 4-methoxyaniline (MOA), IND·MOA, and the salt hemihydrate of IND and 4-phenoxybenzylamine (POBA), IND·POBA·0.5H_2_O, were obtained by solution crystallization from ethanol. In the DSC plots a single thermal event is observed at 104.7 °C (IND·MOA) and 138.5 °C (IND·POBA·0.5H_2_O) corresponding to the melting of the cocrystal and salt (Figures S1 and S2). The X-ray structures are shown in Figures 4 and 5. The C–O bond lengths of the carboxyl group in IND·POBA·0.5H_2_O are 1.243(5) and 1.254(5) Å, indicating that proton transfer to the amino group of POBA has taken place. By contrast, the C–O bond lengths of 1.204(3) and 1.289(3) Å in IND·MOA are in line with a C=O double and a C–OH single bond. The protonation state of the carboxyl group in the cocrystal and salt was confirmed by IR spectroscopy (Figures S3 and S4). The ν(O–H) and ν(C=O) bands of the COOH dimer in the 3350 and 1700 cm^–1^ region have disappeared in the IR spectrum of IND·POBA·0.5H_2_O. Instead, a carbonyl band at 1542 cm^–1^ is observed which is characteristic for a carboxylate salt. By contrast, the carbonyl band at 1669 cm^–1^ in the IR spectrum of IND·MOA is consistent with adduct formation between neutral IND and MOA.

In IND·MOA, each MOA molecule interacts with three IND molecules via NH_2_···O=C [2.929(5) Å], NH_2_···OCH_3_ [3.084(5) Å] and H_2_N···HOOC [2.686(4) Å] hydrogen bonds. In IND·POBA·0.5H_2_O the cation and anion are connected through charge-assisted NH_3_^+^···^–^OOC hydrogen bonds [2.724(5), 2.775(5) and 2.810(5) Å]. The ammonium groups of two POBA cations, a carboxylate group of an IND anion and a carboxylate oxygen of a second IND anion form an  motif. An infinite chain of  rings extends in the *b* direction. In both structures, IND and the coformer are stacked in the direction of the *b* axis.

**Figure 6:**
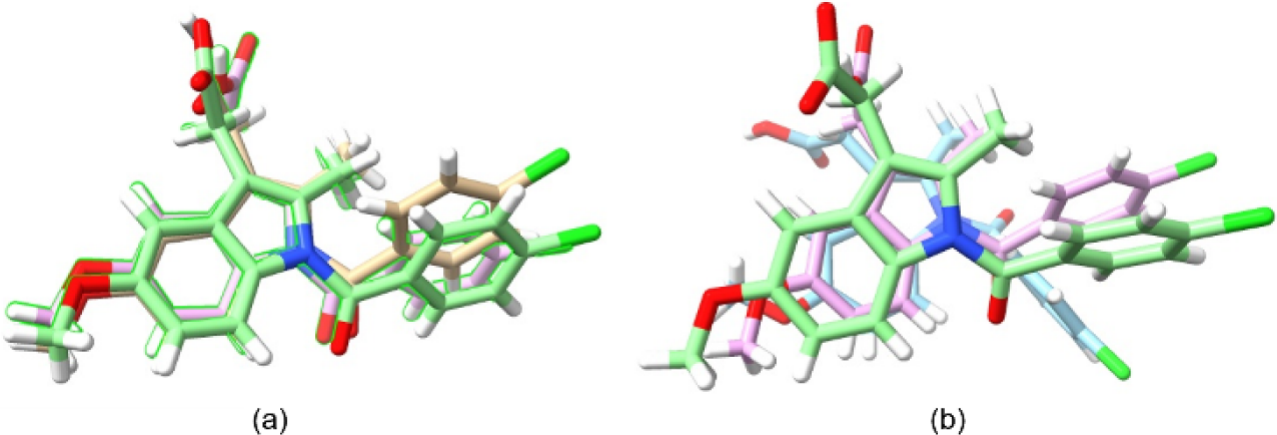
Overlay of the IND molecule in IND·MOA (magenta), and IND·POBA·0.5H_2_O (green) with (a) molecule B (Figure 2) in α-IND (brown) and (b) γ-IND (light blue).

An interesting feature of the α-polymorph of IND is the unusual conformation of one of the symmetry unique IND molecules in the unit cell (*Z*’ = 3). This unusual conformation is characterized by an amide dihedral angle ϕ(C8–N1–C7′–C1′, see Figure 1 for the numbering scheme) of −156.153° compared to 29.352, −27.653, and −37.112° in the other two symmetry unique molecules in α-IND (CSD code INDMET02) and in γ-IND (*Z*’ = 1, CSD code INDMET01). The rotation of the amide dihedral angle by about 180° results in an unusual short distance between the methyl group at C2 and the chlorophenyl ring (C1’···CH_3_ distance = 2.963 Å). Quantum mechanical calculations reported by Ruggiero, Zeitler and coworkers suggested that this conformation presents a high kinetic barrier to the formation of the α-polymorph at room temperature.^26^ Interestingly, a similar conformation with ϕ ∼ 150° and *d*(C1’···CH_3_) ∼ 3 Å is observed in both, IND·MOA and IND·POBA·0.5H_2_O (Table 1, Figure 6).

**Table 1:** IND Conformation in α-IND, γ-IND, IND·MOA, and IND·POBA·0.5H_2_O

	ϕ(C8–N1–C7′–C1’)	*d*(CH_3_–C1’)
α-IND (*Z*’ = 3)^49^	29.35	4.427
	–156.15	2.963
	–27.65	4.425
γ-IND (*Z*’ = 1)^50^	–37.65	4.392
IND·MOA	–151.63	3.043
IND·POBA·0.5H_2_O	–156.98	3.065

### Stacking Interactions and Needle Growth of IND·MOA and IND·POBA·0.5H_2_O

The Hirshfeld surfaces and 2D fingerprint plots for IND·MOA and IND·POBA·0.5H_2_O are shown in Figures S5 and S6. The percentages of H···H, C···H/H···C, O···H/H···O and Cl···H/H···Cl contributions are comparable to those in α-IND (Table 2).

**Table 2:** Percentage Contributions to the Hirshfeld Surface of α-IND, IND·MOA, and IND·POBA·0.5H_2_O

	α-IND	IND·MOA	IND·POBA·0.5H_2_O
H···H	36.4	40.0	38.3
C···H/H···C	21.9	24.7	24.7
O···H/H···O	20.7	20.9	19.9
Cl···H/H···Cl	11.5	9.9	8.2
C···C	1.3	1.4	1.2
other	8.2	3.1	7.7

**Figure 7:**
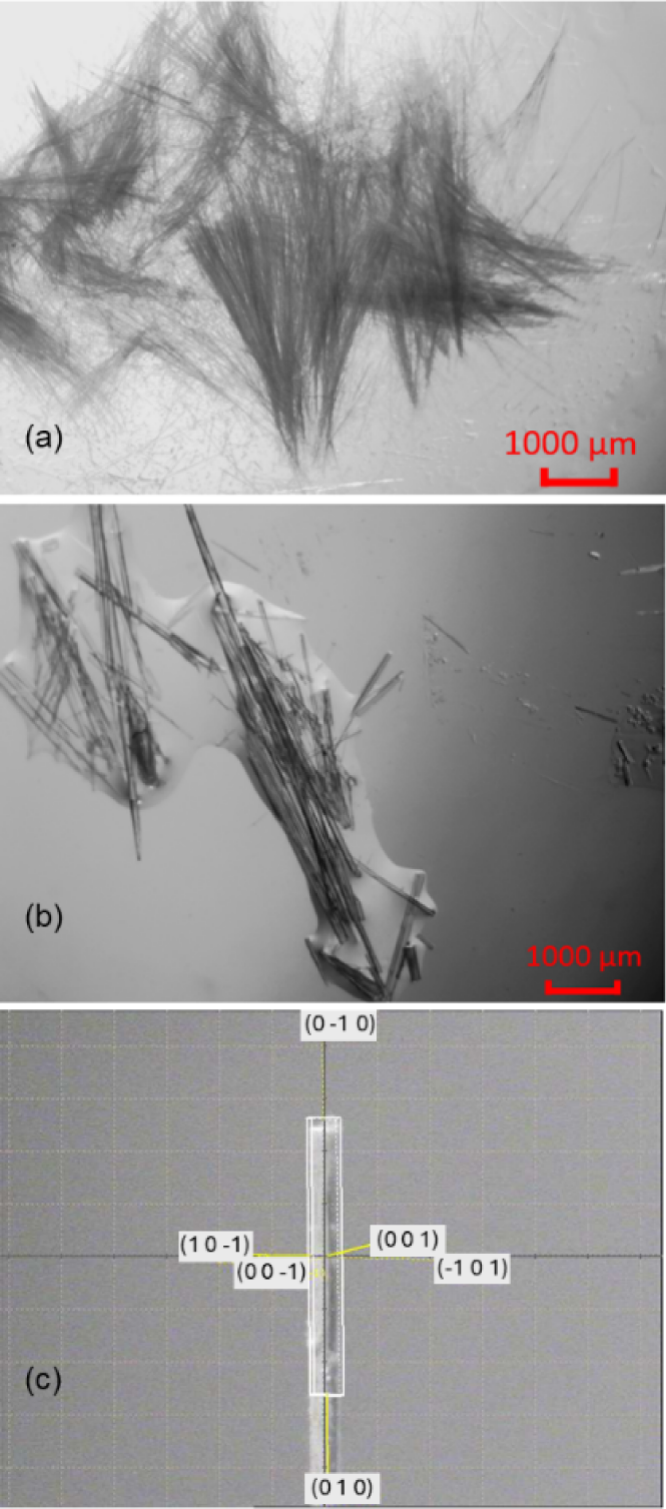
IND·POBA·0.5H_2_O crystals grown from (a) ethyl acetate and (b) ethanol. (c) Face-indexing of an IND·POBA·0.5H_2_O crystal grown from ethanol and mounted on a diffractometer.

The highest total (Pixel) interaction energy of IND·POBA·0.5H_2_O is greater than −400 kJ mol^–1^ (Table S4) and reflects the salt nature of the cocrystallization product. The combination of strong charge-assisted hydrogen bonding and stacking interactions in the structure of IND·POBA·0.5H_2_O promotes growth in the *b* direction leading to the formation of long, extremely thin needles in ethyl acetate (Figure 7a). Needles of sufficient thickness for single crystal X-ray analysis (albeit still relatively thin) were obtained from ethanol (Figure 7b). As shown in Figure 7c, the crystal growth direction is along the shortest axis, following the general rule established in the literature.^51,52^ Crystallization attempts in methanol and acetone resulted in oily residues.

**Figure 8:**
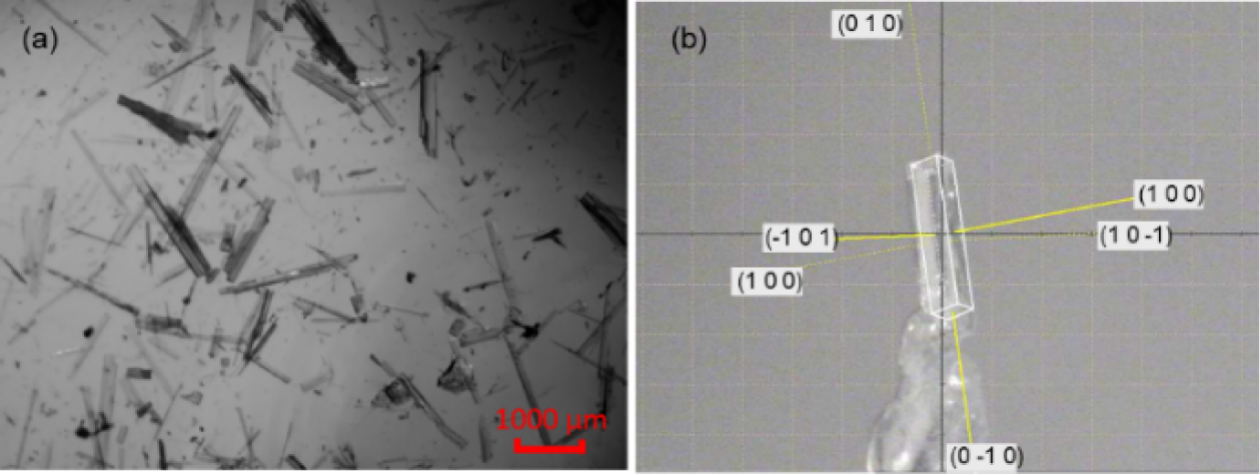
(a) IND·MOA crystals grown from ethanol and (b) face-indexing of an IND·MOA crystal mounted on a diffractometer.

Methanol, acetone, acetonitrile and ethyl acetate did not give crystalline IND·MOA. Like IND·POBA·0.5H_2_O, the IND·MOA crystals grown form ethanolic solution had a needlelike morphology with extended growth along the *b* axis (Figure 8). However, the needles were thicker and shorter compared to IND·POBA·0.5H_2_O.

In a previous paper we have identified van der Waals contact stacking as an important driver of needle growth.^13^ To analyze the stacking interactions and needle growth of IND·MOA and IND·POBA·0.5H_2_O further, the percentage of atoms in van der Waals contact with their stack neighbors was calculated. In the case of IND·MOA, 57% of the IND atoms were found to be in van der Waals contact with atoms of the molecule above and below in the stack. This value is similar to the fraction of atoms in van der Waals contact in α-IND (56, 57 and 60% for the three crystallographically independent molecules). However, within the MOA stacks, a considerably smaller fraction of atoms—only 37% - are in van der Waals contact with stack neighbors. The relative percentages of IND and MOA atoms in van der Waals contact matches the relative Pixel dispersion interaction energies in the stacks. Details of the Pixel calculations for IND·MOA are shown in Table S5. The main contributions to the stability of the IND·MOA cocrystal are van der Waals contact stacking between IND molecules, C–H···π interactions between IND and MOA and COOH···N hydrogen bonding. The strongest interaction is the interaction between stack neighbors in the IND stack. This interaction has a total Pixel energy of −71.8 kJ mol^–1^ with a dispersion component of −70 kJ mol^–1^. COOH···N hydrogen bonding is the second strongest interaction with an *E*_total_ of −49.5 kJ mol^–1^ and an *E*_Coulombic_ of −85.8 kJ mol^–1^. The MOA coformer has stack interactions of only −11.8 kJ mol^–1^ (dispersion contribution −14.3 kJ mol^–1^). The small percentage of atoms in van der Waals contact within the coformer stacks and the resulting low interaction energy explains the less extreme needle growth of the cocrystal compared to α-IND. In IND·POBA·0.5H_2_O, the percentage of IND atoms in van der Waals contact with atoms of their stack neighbors is smaller compared to α-IND and IND·MOA (46% vs 56–60% and 57%, respectively). Within the POBA stacks, the percentage of atoms in van der Waals contact with atoms of neighboring molecules is 34%, even smaller than the fraction in the MOA stacks of IND·MOA. However, in contrast to IND·MOA, the stacking in the salt is supported through strong charge-assisted hydrogen bonding in the stack direction that outweighs the weaker dispersion interaction. As a result IND·POBA·0.5H_2_O forms thinner needles than IND·MOA. We have previously discussed van der Waals contact stacking of nonflat molecules that self-fit, i.e., that fit into each other like stackable bowls or chairs.^13^ The close molecular fitting reflected in a high percentage of atoms in van der Waals contact with atoms of their stack neighbors is in line with Kitaigorodsky’s theory of close packing in organic crystals.^53^

### Co-grinding of IND with MOA and POBA

The mechanochemical synthesis of cocrystals is widely used as a solvent-free alternative to solution crystallization.^54^ Since MOA has a relatively low melting point of only 56–59 °C, we investigated the milling-induced cocrystallization of IND and MOA at low temperature. On cryomilling the equimolar mixture converted to a viscous, gluey material that could not be scraped off the walls of the milling jars. When IND and MOA were milled in a 2:1 ratio, a powdery sample was obtained that was identified by XRPD analysis as a mixture of IND·MOA and excess IND (Figure S7). No milling experiments were performed with POBA which is a liquid at room temperature.

**Figure 9:**
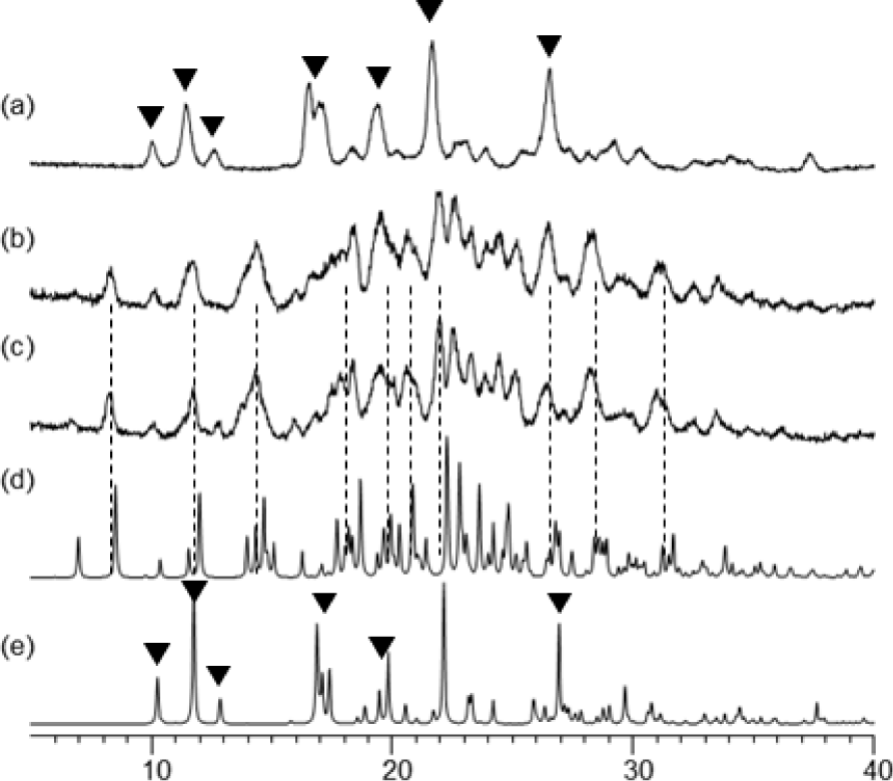
Recrystallization of amorphous IND prepared by milling of γ-IND in the absence and in the presence of MOA. (a) IND milled for 60 min and stored for 5 d at 56% RH. (b) IND milled for 60 min in the presence of 2.5% MOA and stored for 5 d at 56% RH. (c) IND milled for 60 min in the presence of 5% MOA and stored for 5 d at 56% RH. (d) Theoretical pattern of α-IND. (e) Theoretical pattern of γ-IND. (▼) Most characteristic Bragg peaks of γ-IND.

**Figure 10:**
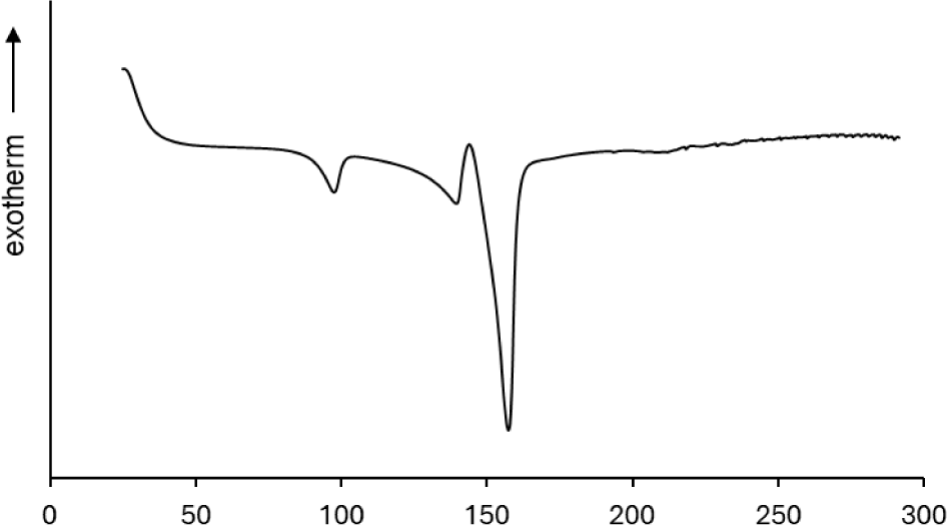
DSC plot of IND milled in the presence of 5% MOA and recrystallized at room temperature and 56% RH.

IND converts to the amorphous state when milled on its own and recrystallizes to the stable γ-form (Figure S8). Bragg peaks of α-IND are not observed during the recrystallization suggesting that the conversion is direct and not via the metastable polymorph. α-IND is known to be stable toward transformation for months^55^ so that the formation of small amounts of transient α-IND below the detection limit is unlikely. The observation that IND·MOA forms on milling and the fact that the IND molecules in the cocrystal are stacked and adopt the unusual conformation of α-IND prompted us to investigate whether traces of MOA affect the recrystallization of amorphous IND. Indeed, when γ-IND was amorphized by milling for 60 min in the presence of 2.5 or 5% MOA, the sample recrystallized to α-IND on storage at 56% RH and room temperature for 5 d (Figure 9). The DSC plot of the recrystallized sample shows an endotherm at 97.7 °C which corresponds to the melting of the cocrystal, an endothermic event with a peak temperature of 139.4 °C which may be assigned to recrystallization of the melt and a major endotherm for the melting of IND (Figure 10). The melting of IND is completed at 158.9 °C, in line with the literature data of the α-form.^22^ The recrystallization to α-IND was also confirmed by IR spectroscopy (Figure S9). The α-polymorph can be clearly identified by its C=O bands at 1731 cm^–1^ (COOH), 1685 cm^–1^ (COOH, amide), and 1654 cm^–1^ (amide), while in the IR spectrum of γ-IND two C=O bands appear at 1714 (COOH) and 1690 cm^–1^ (amide).^22^

Traces of the cocrystal that form during milling may act as templates for the stacked α-polymorph. Alternatively, transient cocrystal formation may generate the α-phase conformation of the IND molecules. It is noteworthy that the α-polymorph usually does not form easily at low temperature, since the unfavorable molecular conformation results in a high energy barrier.^26^

IND is X-ray amorphous after 20 min of milling. However, in this case traces of MOA have no effect on the recrystallization and the sample crystallizes to the γ-polymorph (Figure S10). The sample may still contain traces of crystalline γ-IND that act as seeds or the conversion to the α-phase conformation requires longer milling times in line with the high energy barrier to be overcome.

## Conclusions

The cocrystal IND·MOA and the molecular salt IND·POBA·0.5H_2_O have stacked structures which result in needle morphologies. The interaction energy and thus the needle thickness can be manipulated through the choice of coformer. Specifically, the size of the coformer has an important effect. The smaller coformer MOA the stacks of which have only weak neighbor interactions can block the extreme anisotropic growth of α-IND. Our work thus shows how crystal engineering can be applied to control the needle growth of stacked structures without breaking the stacking preferences of the parent compound. This may find interesting applications in the crystallization of π-stacked functional materials. The observation that the coformer can also be used to alter the recrystallization pathway of amorphous IND can also be exploited in future work. If a similar strategy is applicable to a broader range of systems, this could offer a new route to metastable or elusive polymorphs.

## Supporting Information Available

Additional XRPD patterns; IR spectra; DSC plots; Hirshfeld surface, molecules with atoms, 2D fingerprint plots; sample compositions; crystal data; detailed results of the pixel calculations (PDF)
