# Supplementary material for: Stacking Interactions in Indomethacin Solid-State Forms
Source: Cryst Growth Des. 2025 Mar 10;25(6):1776–84. doi: 10.1021/acs.cgd.4c01507 (PMC11926784; doi:10.1021/acs.cgd.4c01507)
Supplement: Supplementary file 1 — cg4c01507_si_001.pdf [file cg4c01507_si_001.pdf]

Stacking interactions in indomethacin solid-state forms

Nazanin Fereidouni,[1] Marwah Aljohani,[2] and Andrea Erxleben*[1,3]

[1] School of Biological and Chemical Sciences, University of Galway, Galway, H91TK33, Ireland

[2] Department of Chemistry, College of Science, Imam Abdulrahman Bin Faisal University, P.O. Box 76971, Dammam 31441, Saudi Arabia

[3] Synthesis and Solid State Pharmaceutical Centre (SSPC), Limerick, V94 T9PX, Ireland

*Corresponding author email address: andrea.erxleben@nuigalway.ie

Supporting Information

Table S1. Sample compositions in the ball-milling experiments

	Method	IND	MOA	TIME
1	Ball-milling	500 mg	-	60 min
2	Ball-milling	500 mg	12.5 mg	20 min
3	Ball-milling	500 mg	12.5 mg	60 min
4	Ball-milling	500 mg	25 mg	20 min
5	Ball-milling	500 mg	25 mg	60 min
6	Cryomilling	500 mg	86 mg	60 min

Table S2. Crystal data of IND·MOA and IND·POBA·$0.5H_2O$

	IND·MOA	IND·POBA·$0.5H_2O$
Empirical formula	$C_{26}H_{25}ClN_2O_5$	$C_{32}H_{30}ClN_2O_{5.5}$
Formula weight	480.95	566.06
Temperature	293.0(2) K	293.0(2) K
Wavelength	0.71073 Å	0.71073 Å
Crystal system	Monoclinic	Monoclinic
Space group	$P2_1/n$	$P2_1/c$
Unit cell dimensions	a = 21.0263(13) Å	a = 11.3554(12) Å
	b = 5.2856(3) Å	b = 5.9927(7) Å
	c = 21.2330(15) Å	c = 41.259(4) Å
	β = 95.440(6)°	β = 95.063(10)°
Volume	2349.1(3) Å^3	2796.7(5) Å^3
Z	4	4
Density (calculated)	1.360 Mg/m^3	1.344 Mg/m^3
Goodness-of-fit on F^2	1.028	0.990
R_1 [I>2sigma(I)]	0.0580	0.0865
wR_2 (all data)	0.0910	0.1578
Reflections collected	19413	19275
Independent reflections	4623	4915
R(int)	0.0828	0.170

Table S3. Pixel energies (kJ mol^{-1}) of α-IND (Refcode: INDMET02)

	Symmetry operation	Residues	Distance (Å)	Coulombic	Polarisability	Dispersion	Repulsion	Pixel	% vdW
1	1+x, y, z	IND_B…IND_B	5.461	-57.5	-31.2	-69.5	54	-104.1	57.3
2	x, y, z	IND_A…IND_B 2 x OH…O=C	8.381	-107.5	-48.4	-22.6	102.8	-75.8	9.8
3	1+x, y, z	IND_A…IND_A	5.461	-29.9	-16.4	-75.1	56	-65.3	56.1
4	1+x, y, z	IND_C…IND_C	5.461	-40	-19.5	-78	61.9	-75.7	59.8
5	x, y, 1+z	IND_B…IND_C OH…O=C	10.219	-34.9	-15	-16.1	31.6	-34.4	12.2

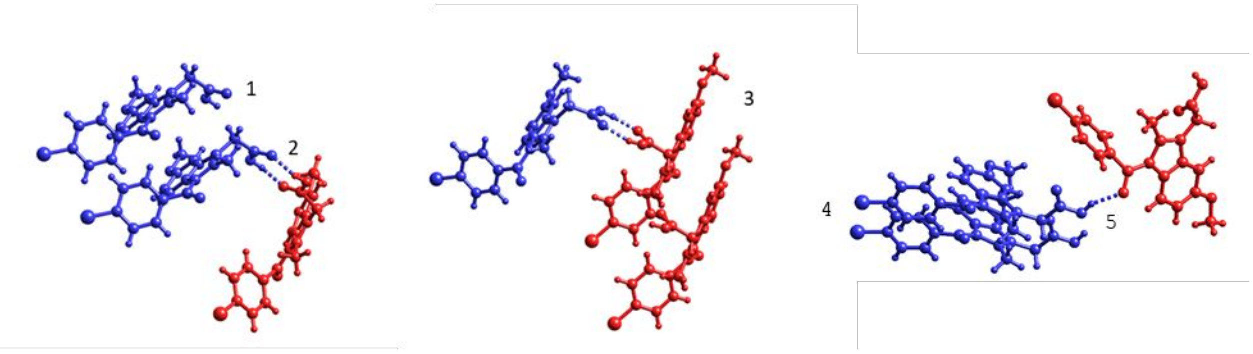

Table S4. Pixel energies (kJ mol^{-1}) of IND·POBA·0.5H_2O

	Symmetry operation	Residues	Distance (Å)	Coulombic	Polarisability	Dispersion	Repulsion	Pixel	% vdW
1	x,y-1, z	IND…POBA	8.488	-409	-68.4	-35.3	44.6	-468.7	35.4
2	-x+1, y-1/5,z+3/2	IND…POBA	10.845	-394.2	-59.5	-12.2	30.4	-435.5	15.8
3	x,y,z	IND…POBA	6.994	-332	-68.6	-30.1	47.1	-383.7	33.7

Table S5. Pixel energies (kJ mol^{-1}) of IND·MOA

	Symmetry operation	Residues	Distance (Å)	Coulombic	Polarisability	Dispersion	Repulsion	Pixel	% vdW
1	x, 1+y, z	IND…IND	5.286	-32.5	-11.3	-70	42	-71.8	57.3
2	x, y, z	IND…MOA	7.305	-85.8	-33.5	-18.5	88.3	-49.5	20.3
3	1-x, 1-y, 1-z	IND…MOA	6.007	-25.9	-10.8	-33.1	26.9	-42.9	33.7
4	x, 1+y, z	MOA…MOA	5.286	-3.6	-2.9	-14.3	9	-11.8	37.5

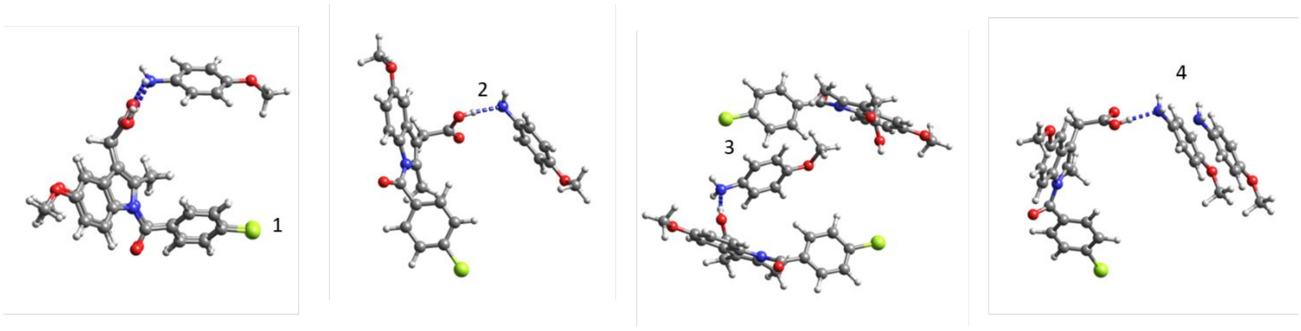

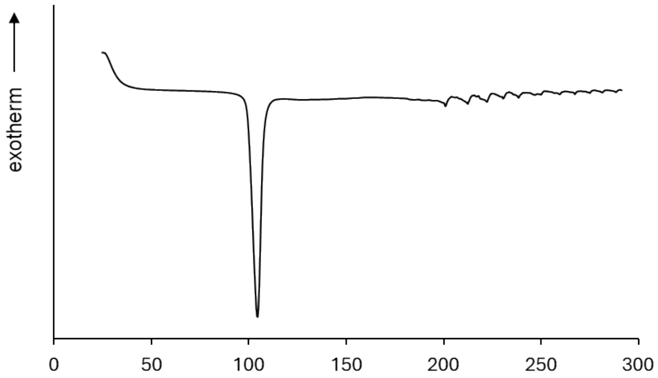

Fig. S1 DSC plot of IND·MOA.

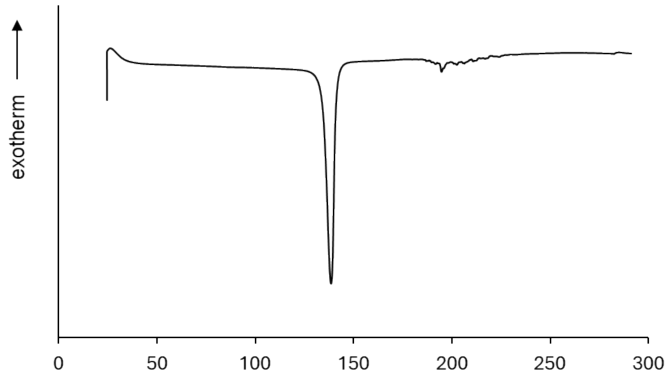

Fig. S2 DSC plot of IND·POBA·0.5H_2O.

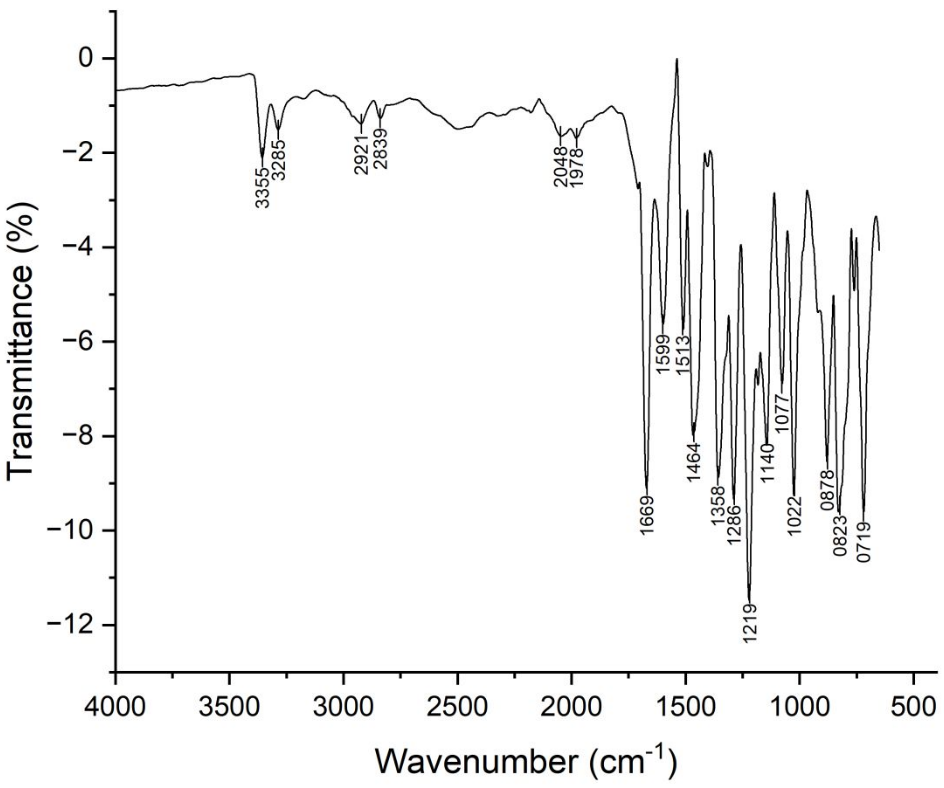

Fig. S3 IR spectrum of IND·MOA.

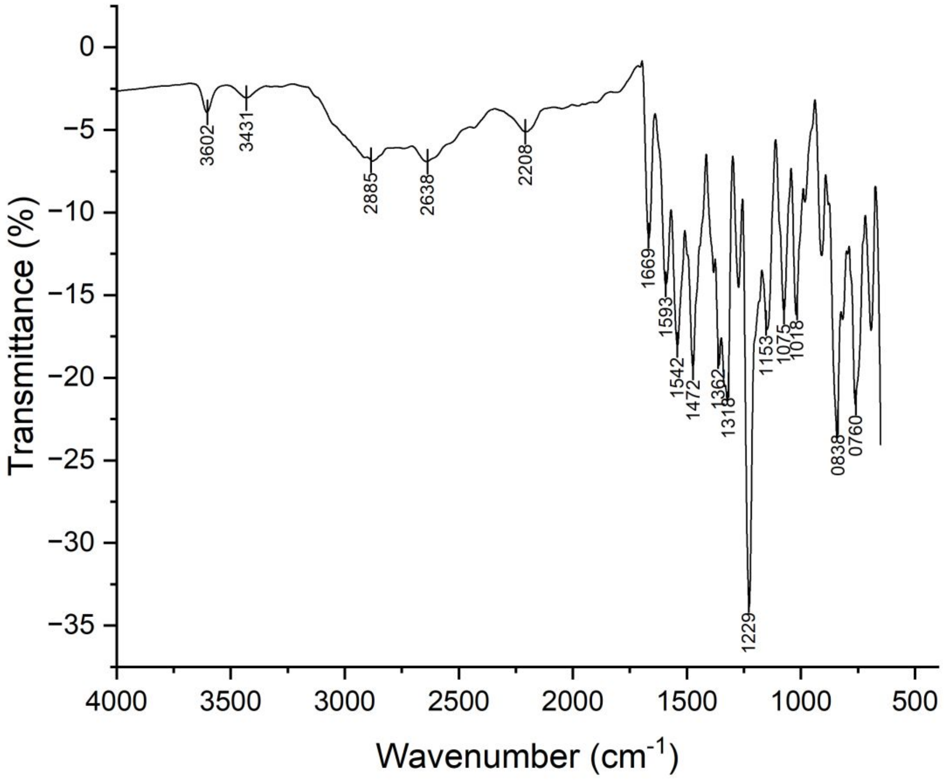

Fig. S4 IR spectrum of IND·POBA·0.5H_2O.

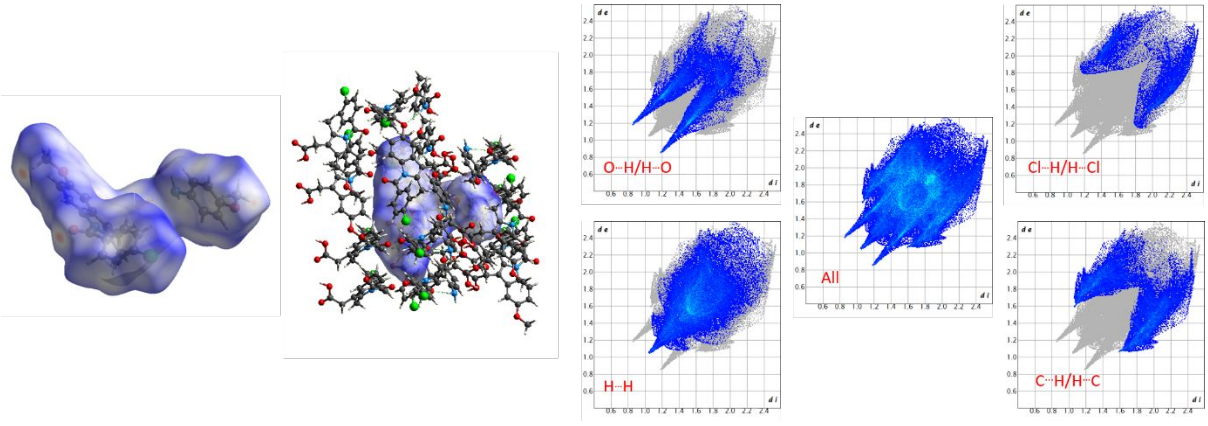

(a) (b) (c)

Fig. S5 (a) Hirshfeld surface (d_{norm}) of IND·MOA. The red spots represent hydrogen bonding. (b) Molecules with atoms within a distance of 3.8 Å outside the Hirshfeld surface map and (c) 2D fingerprint plots (d_i vs d_e, where d_i and d_e are the distances to the nearest atom inside and outside the surface, respectively).

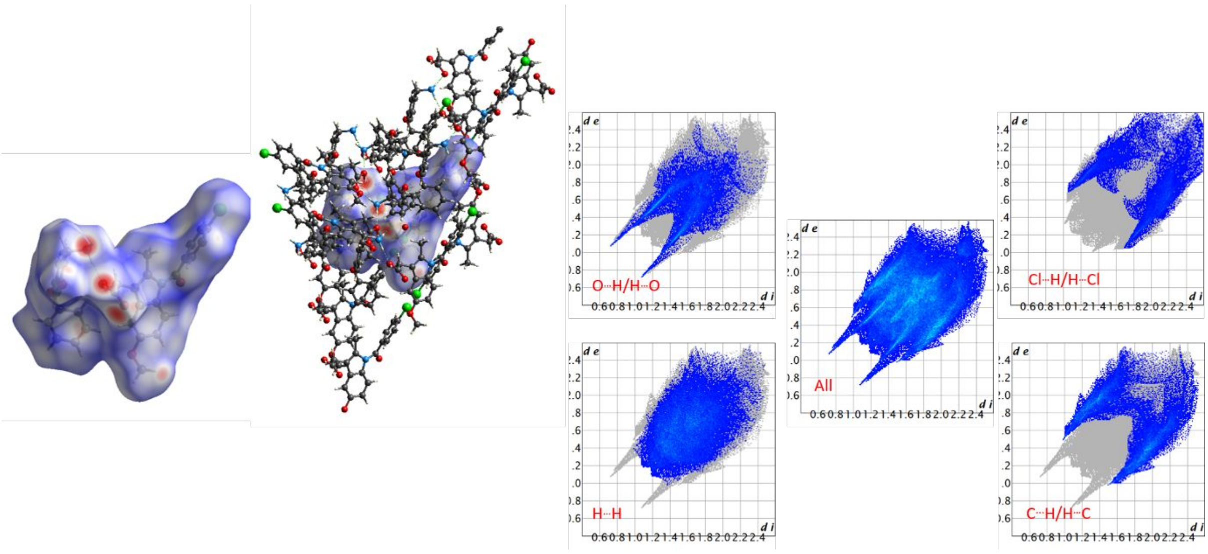

(a) (b) (c)

Fig. S6 (a) Hirshfeld surface (d_{norm}) of IND·POBA·0.5H_2O. The red spots represent hydrogen bonding. (b) Molecules with atoms within a distance of 3.8 Å outside the Hirshfeld surface map and (c) 2D fingerprint plots (d_i vs d_e, where d_i and d_e are the distances to the nearest atom inside and outside the surface, respectively).

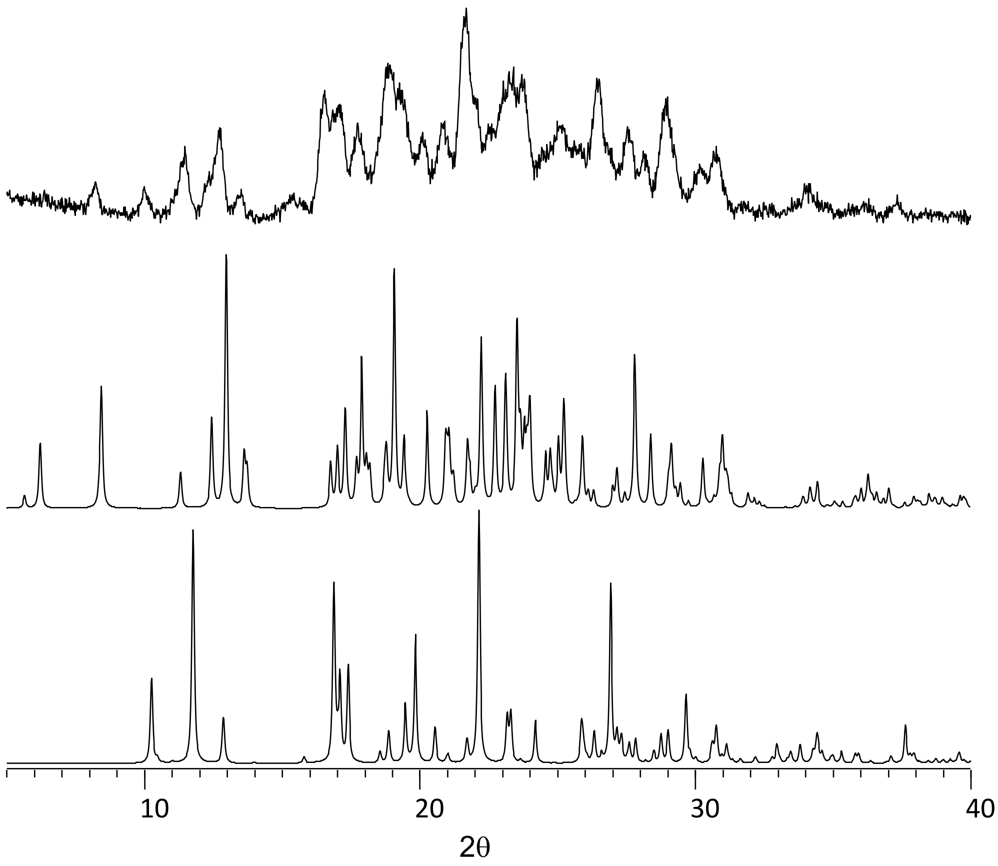

Fig. S7 Top: XRPD pattern of a 2:1 mixture of IND and MOA after cryomilling for 60 min. Middle: Theoretical XRPD pattern of IND·MOA calculated from the single crystal data. Bottom: Theoretical XRPD pattern of γ-IND calculated from the single crystal data (INDMET03).

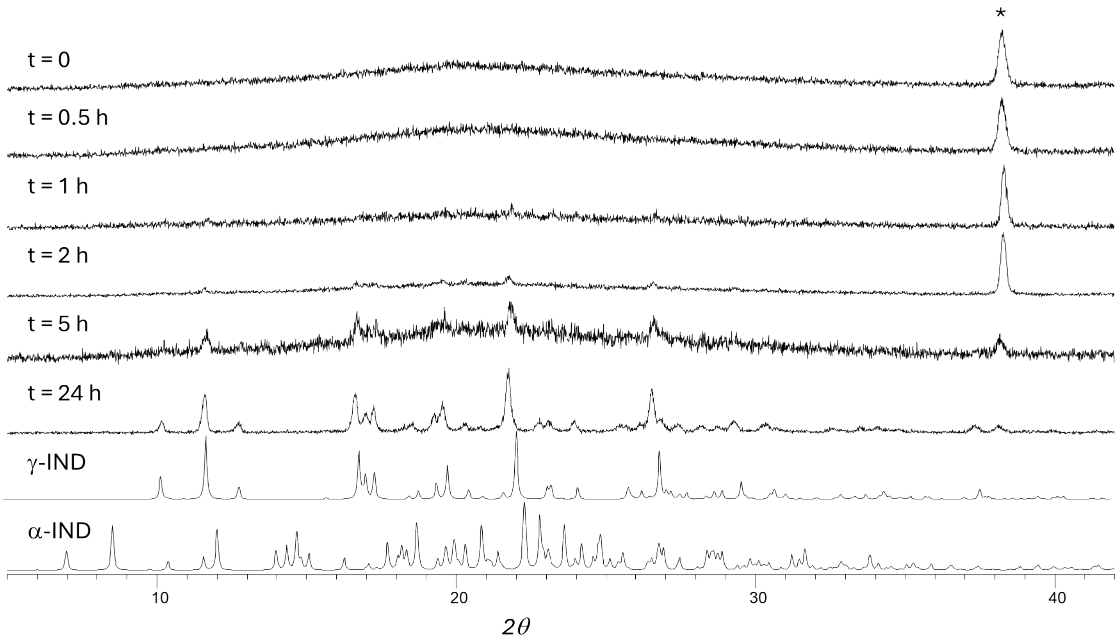

Fig. S8. Recrystallization of amorphous IND prepared by milling of γ-IND for 60 min at room temperature: XRPD patterns at different time points after milling and theoretical XRPD patterns of α- and γ-IND calculated from the single crystal data (INDMET02 and INDMET03). *Diffraction peak from the aluminium sample holder.

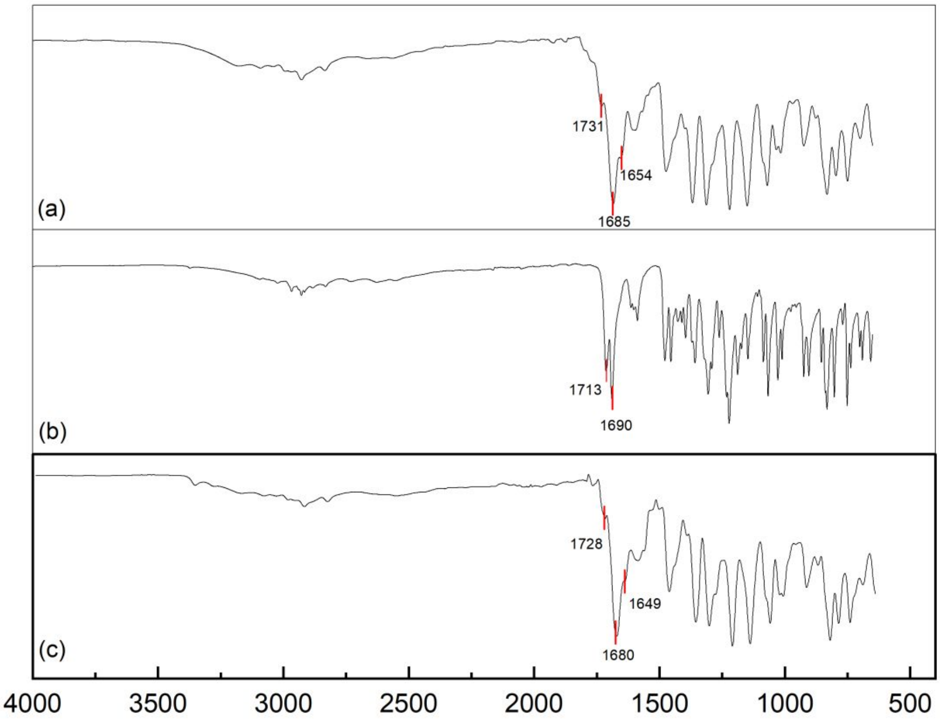

Fig. S9 IR spectrum of (a) α-IND (prepared according to X. Chen, K. R. Morris, U. J. Griesser, S. R. Byrn and J. G. Stowell, *J. Am. Chem. Soc.*, 2002, 124, 15012–15019), (b) commercial γ-IND and (c) recrystallized sample of α-IND milled in the presence of 5 % MOA.

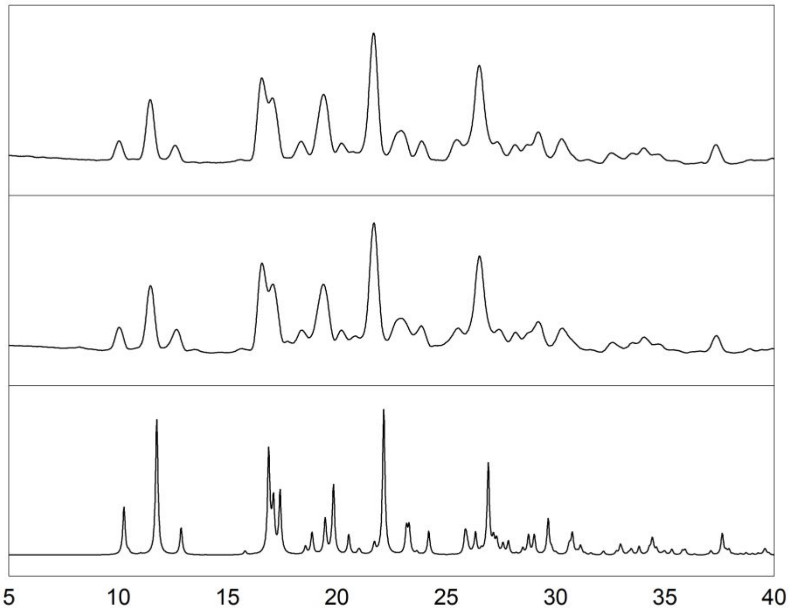

Fig. S10 Top: XRPD pattern of a recrystallized sample of amorphous IND prepared by milling of γ-IND in the presence of 2.5 % MOA for 20 min at room temperature. Middle: XRPD pattern of a recrystallized sample of amorphous IND prepared by milling of γ-IND in the presence of 5 % MOA for 20 min at room temperature. Bottom: Theoretical XRPD pattern of γ-IND calculated from the single crystal data (INDMET03).
